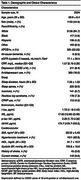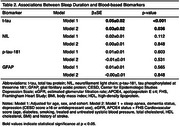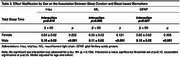# Sex differences in the association between sleep duration and blood‐based biomarkers of Alzheimer's disease and neurodegeneration: The Framingham Heart Study

**DOI:** 10.1002/alz70856_099558

**Published:** 2025-12-24

**Authors:** Vanessa M. Young, Crystal Wiedner, Andrée‐Ann Baril, Christopher R. Frei, Antonio L Teixeira, Agustin Ruiz, Matthew P. Pase, Alexa S Beiser, Jayandra Jung Himali, Sudha Seshadri

**Affiliations:** ^1^ Glenn Biggs Institute for Alzheimer's & Neurodegenerative Diseases, University of Texas Health Science Center, San Antonio, TX, USA; ^2^ Graduate School of Biomedical Sciences, University of Texas Health Science Center, San Antonio, TX, USA; ^3^ Glenn Biggs Institute for Alzheimer's & Neurodegenerative Diseases, University of Texas Health Science Center, San Antonio, TX, USA; ^4^ Framingham Heart Study, Framingham, MA, USA; ^5^ Center for Advanced Research in Sleep Medicine, Hôpital du Sacré‐Coeur de Montréal, CIUSSS‐NIM, Montreal, QC, Canada; ^6^ Université de Montréal, Montreal, QC, Canada; ^7^ College of Pharmacy, The University of Texas at Austin, Austin, TX, USA; ^8^ School of Medicine, University of Texas Health Science Center at San Antonio, San Antonio, TX, USA; ^9^ Research Center and Memory Clinic, Ace Alzheimer Center Barcelona, Universidad Internacional de Catalunya, Barcelona, Barcelona, Spain; ^10^ Department of Microbiology, Immunology and Molecular Genetics, University of Texas Health Science Center, San Antonio, TX, USA; ^11^ Biomedical Research Networking Centre in Neurodegenerative Diseases (CIBERNED), National Institute of Health Carlos III, Madrid, Madrid, Spain; ^12^ Monash University, Clayton, VIC, Australia; ^13^ The Framingham Heart Study, Framingham, MA, USA; ^14^ Boston University School of Public Health, Boston, MA, USA; ^15^ Boston University Chobanian & Avedisian School of Medicine, Boston, MA, USA; ^16^ Department of Population Health Sciences, University of Texas Health Sciences Center, San Antonio, TX, USA; ^17^ Glenn Biggs Institute for Alzheimer's & Neurodegenerative Diseases, University of Texas Health San Antonio, San Antonio, TX, USA; ^18^ Department of Neurology, Boston University School of Medicine, Boston, MA, USA

## Abstract

**Background:**

Sleep disturbances are associated with inflammatory and neurodegenerative processes, as well as greater Alzheimer's disease (AD) risk. However, the relationship between sleep duration and blood‐based biomarkers (BBMs) of neurodegeneration and AD pathology remains understudied, particularly across sexes. In a community‐based cohort, we examined the cross‐sectional associations between self‐reported sleep duration and BBMs of neurodegeneration (t‐tau, NfL), AD pathology (*p*‐tau‐181), and astrocyte activation (GFAP), and explored potential sex‐specific effects.

**Methods:**

Our sample included 2,254 participants (mean age 69.9±8.4 years, 44.9% male) from the Framingham Heart Study Offspring (exam 9) and Omni 1 (exam 4) cohorts who completed their visits between 2011‐2014. Sleep duration was assessed by a technician‐administered questionnaire. Plasma t‐tau, *p*‐tau‐181, GFAP, and serum NfL were measured using Single Molecule Array (SIMOA) assays. All BBMs were log‐transformed and standardized for analysis. Model 1 adjusted for age, sex‐at‐birth, and cohort; Model 2 additionally adjusted for sleep apnea, cardiovascular risk factors, depression (defined as Center for Epidemiologic Studies Depression Scale ≥16 or antidepressant use), eGFR, APOE4, stroke, and all‐cause‐dementia.

**Results:**

Table 1 depicts sample characteristics. Longer sleep duration was associated with higher t‐tau levels across all models (Model 1: β±SE=0.05±0.02, *p* <0.001; Model 2: 0.03±0.02, *p* = 0.036) (Table 2). There were statistically significant sex interactions for t‐tau (*p* = 0.019), NfL (*p* = 0.006), and GFAP (*p* = 0.007). In sex‐stratified analyses (Table 3), we observed positive associations between longer sleep duration and higher biomarker levels (t‐tau: 0.10±0.02, NfL: 0.11±0.02, GFAP: 0.010±0.02; all *p* <0.001) in males but not in females. No significant associations or interactions were observed for *p*‐tau‐181.

**Conclusion:**

Sex significantly modified associations between sleep duration and BBMs. Specifically, only males showed significant positive associations between longer sleep and elevated t‐tau, NfL, and GFAP levels. Relative to females, males have heightened inflammatory responses to sleep disturbances, which may partially explain our sex‐specific findings with BBMs of neuronal injury and astrogliosis. Although limited by cross‐sectional design and self‐reported sleep measures, the findings underscore the relevance of sex‐specific analyses in sleep and neurodegeneration research. We will next explore non‐linear associations as short and long sleep may relate to BBMs level differently. Future longitudinal studies with objective sleep measurements are needed.